# Correction: Interactive Effects of Black-Tailed Prairie Dogs and Cattle on Shrub Encroachment in a Desert Grassland Ecosystem

**DOI:** 10.1371/journal.pone.0157688

**Published:** 2016-06-09

**Authors:** The PLOS ONE Staff

## Notice of Republication

This article was republished on June 3, 2016 to correct poor figure quality in the PDF version, which was introduced during the typesetting process. The publisher apologizes for these issues with the figure quality. Please download this article again to view the correct version. The originally published, uncorrected article and the republished, corrected article are provided here for reference.

## Supporting Information

S1 File Originally published, uncorrected article. (PDF)

S2 File Republished, corrected article. (PDF)

The following information is missing from the Acknowledgments: This work was submitted in partial fulfillment of the requirements of the Doctorado en Ciencias Biológicas—Universidad Nacional Autónoma de México (UNAM).
