# Supplementary material for: Interactive Effects of Black-Tailed Prairie Dogs and Cattle on Shrub Encroachment in a Desert Grassland Ecosystem
Source: PLoS One. 2016 May 4;11(5):e0154748. doi: 10.1371/journal.pone.0154748 (PMC4856282; doi:10.1371/journal.pone.0154748)
Supplement: S1 Table — Number of mesquite shrubs (ind/plot and ind/hectare) observed in 2006 and 2011. (DOCX) [file pone.0154748.s001.docx]

**S1. Mesquite abundance database**. Number of mesquite plants (ind/plot and ind/hectare) observed in 2006 and 2011. +P-C = prairie dogs only occurred; +P+C = prairie dogs and cattle occurred together; -P+C = cattle only occurred; -P-C = both prairie dog and cattle were absent.

| **Year** | **Plot** | **Treatment** | **Mesquite abundance ind / plot** | **Mesquite abundance ind / ha** |
| --- | --- | --- | --- | --- |
| 2006 | 1 | +P -C | 6 | 16.7 |
| 2006 | 2 | +P -C | 2 | 5.6 |
| 2006 | 3 | +P -C | 0 | 0.0 |
| 2006 | 4 | +P -C | 4 | 11.1 |
| 2006 | 1 | -P -C | 1 | 2.8 |
| 2006 | 2 | -P -C | 5 | 13.9 |
| 2006 | 3 | -P -C | 6 | 16.7 |
| 2006 | 4 | -P -C | 3 | 8.3 |
| 2006 | 1 | -P +G | 3 | 8.3 |
| 2006 | 2 | -P +G | 0 | 0.0 |
| 2006 | 3 | -P +G | 2 | 5.6 |
| 2006 | 4 | -P +G | 3 | 8.3 |
| 2006 | 1 | +P +G | 0 | 0.0 |
| 2006 | 2 | +P +G | 2 | 5.6 |
| 2006 | 3 | +P +G | 5 | 13.9 |
| 2006 | 4 | +P +G | 0 | 0.0 |
| 2011 | 1 | +P -C | 8 | 22.2 |
| 2011 | 2 | +P -C | 3 | 8.3 |
| 2011 | 3 | +P -C | 0 | 0.0 |
| 2011 | 4 | +P -C | 4 | 11.1 |
| 2011 | 1 | -P -C | 11 | 30.6 |
| 2011 | 2 | -P -C | 11 | 30.6 |
| 2011 | 3 | -P -C | 23 | 63.9 |
| 2011 | 4 | -P -C | 10 | 27.8 |
| 2011 | 1 | -P +G | 7 | 19.4 |
| 2011 | 2 | -P +G | 5 | 13.9 |
| 2011 | 3 | -P +G | 6 | 16.7 |
| 2011 | 4 | -P +G | 7 | 19.4 |
| 2011 | 1 | +P +G | 2 | 5.6 |
| 2011 | 2 | +P +G | 2 | 5.6 |
| 2011 | 3 | +P +G | 5 | 13.9 |
| 2011 | 4 | +P +G | 2 | 5.6 |
