# Supplementary material for: Interactive Effects of Black-Tailed Prairie Dogs and Cattle on Shrub Encroachment in a Desert Grassland Ecosystem
Source: PLoS One. 2016 May 4;11(5):e0154748. doi: 10.1371/journal.pone.0154748 (PMC4856282; doi:10.1371/journal.pone.0154748)
Supplement: S2 Table — Mesquite shrubs observed and measured in 2011. (DOCX) [file pone.0154748.s002.docx]

**S2. Mesquite height and canopy cover database.** Mesquite plants observed and measured in 2011. +P-C = prairie dogs only occurred; +P+C = prairie dogs and cattle occurred together; -P+C = cattle only occurred; -P-C = both prairie dog and cattle were absent.

| **I.D.** | **Plot** | **Treatment** | **Height (cm)** | **Canopy cover (m^2^)** |
| --- | --- | --- | --- | --- |
| 1 | 1 | -P -C | 40 | 10 |
| 2 | 1 | -P -C | 47 | 6 |
| 3 | 1 | -P -C | 40 | 17.6 |
| 4 | 1 | -P -C | 25 | 12 |
| 5 | 2 | -P -C | 50 | 10.6 |
| 6 | 2 | -P -C | 40 | 4.2 |
| 7 | 2 | -P -C | 90 | 136.5 |
| 8 | 2 | -P -C | 50 | 97.2 |
| 9 | 2 | -P -C | 70 | 124.2 |
| 10 | 2 | -P -C | 70 | 32.4 |
| 11 | 3 | -P -C | 65 | 9.6 |
| 12 | 3 | -P -C | 30 | 32.3 |
| 13 | 3 | -P -C | 40 | 21 |
| 14 | 3 | -P -C | 50 | 10.4 |
| 15 | 3 | -P -C | 70 | 20.8 |
| 16 | 3 | -P -C | 36 | 14.3 |
| 17 | 3 | -P -C | 34 | 33 |
| 18 | 3 | -P -C | 30 | 9.6 |
| 19 | 3 | -P -C | 50 | 13.65 |
| 20 | 3 | -P -C | 70 | 19.5 |
| 21 | 3 | -P -C | 50 | 14.85 |
| 22 | 3 | -P -C | 40 | 14.3 |
| 23 | 3 | -P -C | 60 | 28.6 |
| 24 | 3 | -P -C | 50 | 19.5 |
| 25 | 3 | -P -C | 30 | 8 |
| 26 | 3 | -P -C | 35 | 18.2 |
| 27 | 3 | -P -C | 50 | 13.2 |
| 28 | 3 | -P -C | 55 | 9 |
| 29 | 3 | -P -C | 35 | 34.2 |
| 30 | 3 | -P -C | 30 | 9 |
| 31 | 3 | -P -C | 45 | 9.1 |
| 32 | 3 | -P -C | 45 | 14 |
| 33 | 3 | -P -C | 45 | 18.7 |
| 34 | 4 | -P -C | 87 | 9.48 |
| 35 | 4 | -P -C | 20 | 2.88 |
| 36 | 4 | -P -C | 23 | 4.56 |
| 37 | 4 | -P -C | 15 | 2.5 |
| 38 | 4 | -P -C | 30 | 2.75 |
| 39 | 1 | -P +C | 40 | 9.9 |
| 40 | 1 | -P +C | 40 | 6.38 |
| 41 | 1 | -P +C | 56 | 8.8 |
| 42 | 2 | -P +C | 44 | 7.84 |
| 43 | 2 | -P +C | 40 | 20.5 |
| 44 | 2 | -P +C | 43 | 3.74 |
| 45 | 2 | -P +C | 50 | 13.485 |
| 46 | 2 | -P +C | 25 | 2.8 |
| 47 | 3 | -P +C | 80 | 30 |
| 48 | 3 | -P +C | 40 | 10 |
| 49 | 3 | -P +C | 40 | 11.2 |
| 50 | 3 | -P +C | 18 | 10 |
| 51 | 3 | -P +C | 60 | 13.2 |
| 52 | 3 | -P +C | 25 | 4.8 |
| 53 | 4 | -P +C | 40 | 2.7 |
| 54 | 4 | -P +C | 50 | 15.4 |
| 55 | 4 | -P +C | 55 | 9.9 |
| 56 | 1 | +P -C | 56 | 17.6 |
| 57 | 1 | +P -C | 30 | 3.5 |
| 58 | 1 | +P -C | 60 | 13.2 |
| 59 | 1 | +P -C | 46 | 13 |
| 60 | 1 | +P -C | 20 | 3.6 |
| 61 | 1 | +P -C | 30 | 1.8 |
| 62 | 1 | +P -C | 15 | 1.2 |
| 63 | 2 | +P -C | 51 | 20.25 |
| 64 | 2 | +P -C | 61 | 16.104 |
| 65 | 2 | +P -C | 33 | 4.27 |
| 66 | 4 | +P -C | 46 | 6 |
| 67 | 4 | +P -C | 30 | 11.7 |
| 68 | 4 | +P -C | 45 | 20.4 |
| 69 | 4 | +P -C | 35 | 2.4 |
| 70 | 1 | +P +C | 60 | 0.9 |
| 71 | 1 | +P +C | 35 | 3 |
| 72 | 2 | +P +C | 10 | 1.024 |
| 73 | 2 | +P +C | 3 | 0.728 |
| 74 | 2 | +P +C | 45 | 33.32 |
| 75 | 3 | +P +C | 35 | 12 |
| 76 | 3 | +P +C | 20 | 12 |
| 77 | 3 | +P +C | 15 | 10.8 |
| 78 | 3 | +P +C | 9 | 0.837 |
| 79 | 4 | +P +C | 10 | 3.5 |
| 80 | 4 | +P +C | 30 | 6 |
