# Supplementary material for: Interactive Effects of Black-Tailed Prairie Dogs and Cattle on Shrub Encroachment in a Desert Grassland Ecosystem
Source: PLoS One. 2016 May 4;11(5):e0154748. doi: 10.1371/journal.pone.0154748 (PMC4856282; doi:10.1371/journal.pone.0154748)
Supplement: S3 Table — Number of prairie dogs observed (ind/plot and ind/hectare) from 2006 to 2011. (DOCX) [file pone.0154748.s003.docx]

**S3. Prairie dog database**. Number of prairie dogs observed (ind/plot and ind/hectare) from 2006 to 2011. +P-C = prairie dogs only occurred; +P+C = prairie dogs and cattle occurred together; -P+C = cattle only occurred; -P-C = both prairie dog and cattle were absent.

| **Year** | **Treatment** | **Plot** | **Prairie dog abundance (ind / plot)** | **Prairie dog abundance (ind / ha)** |
| --- | --- | --- | --- | --- |
|  |  |  |  |  |
| Spring 2006 | -P -C | 1 | 0 | 0 |
| Spring 2006 | -P -C | 2 | 1 | 3 |
| Spring 2006 | -P -C | 3 | 1 | 3 |
| Spring 2006 | -P -C | 4 | 6 | 17 |
| Spring 2006 | -P +C | 1 | 4 | 11 |
| Spring 2006 | -P +C | 2 | 1 | 3 |
| Spring 2006 | -P +C | 3 | 9 | 25 |
| Spring 2006 | -P +C | 4 | 4 | 11 |
| Spring 2006 | +P -C | 1 | 7 | 19 |
| Spring 2006 | +P -C | 2 | 3 | 8 |
| Spring 2006 | +P -C | 3 | 1 | 3 |
| Spring 2006 | +P -C | 4 | 6 | 17 |
| Spring 2006 | +P +C | 1 | 8 | 22 |
| Spring 2006 | +P +C | 2 | 6 | 17 |
| Spring 2006 | +P +C | 3 | 3 | 8 |
| Spring 2006 | +P +C | 4 | 3 | 8 |
| Fall 2006 | -P -C | 1 | 2 | 6 |
| Fall 2006 | -P -C | 2 | 7 | 19 |
| Fall 2006 | -P -C | 3 | 0 | 0 |
| Fall 2006 | -P -C | 4 | 6 | 17 |
| Fall 2006 | -P +C | 1 | 6 | 17 |
| Fall 2006 | -P +C | 2 | 5 | 14 |
| Fall 2006 | -P +C | 3 | 0 | 0 |
| Fall 2006 | -P +C | 4 | 2 | 6 |
| Fall 2006 | +P -C | 1 | 2 | 6 |
| Fall 2006 | +P -C | 2 | 0 | 0 |
| Fall 2006 | +P -C | 3 | 0 | 0 |
| Fall 2006 | +P -C | 4 | 4 | 11 |
| Fall 2006 | +P +C | 1 | 8 | 22 |
| Fall 2006 | +P +C | 2 | 6 | 17 |
| Fall 2006 | +P +C | 3 | 1 | 3 |
| Fall 2006 | +P +C | 4 | 0 | 0 |
| Spring 2007 | -P -C | 1 | 6 | 17 |
| Spring 2007 | -P -C | 2 | 2 | 6 |
| Spring 2007 | -P -C | 3 | 2 | 6 |
| Spring 2007 | -P -C | 4 | 6 | 17 |
| Spring 2007 | -P +C | 1 | 5 | 14 |
| Spring 2007 | -P +C | 2 | 11 | 31 |
| Spring 2007 | -P +C | 3 | 1 | 3 |
| Spring 2007 | -P +C | 4 | 3 | 8 |
| Spring 2007 | +P -C | 1 | 3 | 8 |
| Spring 2007 | +P -C | 2 | 5 | 14 |
| Spring 2007 | +P -C | 3 | 0 | 0 |
| Spring 2007 | +P -C | 4 | 3 | 8 |
| Spring 2007 | +P +C | 1 | 25 | 69 |
| Spring 2007 | +P +C | 2 | 32 | 89 |
| Spring 2007 | +P +C | 3 | 2 | 6 |
| Spring 2007 | +P +C | 4 | 9 | 25 |
| Fall 2007 | -P -C | 1 | 0 | 0 |
| Fall 2007 | -P -C | 2 | 0 | 0 |
| Fall 2007 | -P -C | 3 | 0 | 0 |
| Fall 2007 | -P -C | 4 | 4 | 11 |
| Fall 2007 | -P +C | 1 | 2 | 6 |
| Fall 2007 | -P +C | 2 | 1 | 3 |
| Fall 2007 | -P +C | 3 | 0 | 0 |
| Fall 2007 | -P +C | 4 | 0 | 0 |
| Fall 2007 | +P -C | 1 | 3 | 8 |
| Fall 2007 | +P -C | 2 | 4 | 11 |
| Fall 2007 | +P -C | 3 | 8 | 22 |
| Fall 2007 | +P -C | 4 | 13 | 36 |
| Fall 2007 | +P +C | 1 | 12 | 33 |
| Fall 2007 | +P +C | 2 | 16 | 44 |
| Fall 2007 | +P +C | 3 | 17 | 47 |
| Fall 2007 | +P +C | 4 | 5 | 14 |
| Spring 2008 | -P -C | 1 | 0 | 0 |
| Spring 2008 | -P -C | 2 | 0 | 0 |
| Spring 2008 | -P -C | 3 | 0 | 0 |
| Spring 2008 | -P -C | 4 | 0 | 0 |
| Spring 2008 | -P +C | 1 | 0 | 0 |
| Spring 2008 | -P +C | 2 | 0 | 0 |
| Spring 2008 | -P +C | 3 | 0 | 0 |
| Spring 2008 | -P +C | 4 | 0 | 0 |
| Spring 2008 | +P -C | 1 | 6 | 17 |
| Spring 2008 | +P -C | 2 | 19 | 53 |
| Spring 2008 | +P -C | 3 | 15 | 42 |
| Spring 2008 | +P -C | 4 | 10 | 28 |
| Spring 2008 | +P +C | 1 | 18 | 50 |
| Spring 2008 | +P +C | 2 | 31 | 86 |
| Spring 2008 | +P +C | 3 | 26 | 72 |
| Spring 2008 | +P +C | 4 | 7 | 19 |
| Fall 2008 | -P -C | 1 | 0 | 0 |
| Fall 2008 | -P -C | 2 | 0 | 0 |
| Fall 2008 | -P -C | 3 | 0 | 0 |
| Fall 2008 | -P -C | 4 | 0 | 0 |
| Fall 2008 | -P +C | 1 | 0 | 0 |
| Fall 2008 | -P +C | 2 | 0 | 0 |
| Fall 2008 | -P +C | 3 | 0 | 0 |
| Fall 2008 | -P +C | 4 | 0 | 0 |
| Fall 2008 | +P -C | 1 | 1 | 3 |
| Fall 2008 | +P -C | 2 | 2 | 6 |
| Fall 2008 | +P -C | 3 | 0 | 0 |
| Fall 2008 | +P -C | 4 | 2 | 6 |
| Fall 2008 | +P +C | 1 | 7 | 19 |
| Fall 2008 | +P +C | 2 | 6 | 17 |
| Fall 2008 | +P +C | 3 | 4 | 11 |
| Fall 2008 | +P +C | 4 | 2 | 6 |
| Spring 2009 | -P -C | 1 | 0 | 0 |
| Spring 2009 | -P -C | 2 | 0 | 0 |
| Spring 2009 | -P -C | 3 | 0 | 0 |
| Spring 2009 | -P -C | 4 | 0 | 0 |
| Spring 2009 | -P +C | 1 | 0 | 0 |
| Spring 2009 | -P +C | 2 | 0 | 0 |
| Spring 2009 | -P +C | 3 | 0 | 0 |
| Spring 2009 | -P +C | 4 | 0 | 0 |
| Spring 2009 | +P -C | 1 | 3 | 8 |
| Spring 2009 | +P -C | 2 | 4 | 11 |
| Spring 2009 | +P -C | 3 | 5 | 14 |
| Spring 2009 | +P -C | 4 | 2 | 6 |
| Spring 2009 | +P +C | 1 | 16 | 44 |
| Spring 2009 | +P +C | 2 | 14 | 39 |
| Spring 2009 | +P +C | 3 | 19 | 53 |
| Spring 2009 | +P +C | 4 | 2 | 6 |
| Fall 2009 | -P -C | 1 | 0 | 0 |
| Fall 2009 | -P -C | 2 | 0 | 0 |
| Fall 2009 | -P -C | 3 | 0 | 0 |
| Fall 2009 | -P -C | 4 | 0 | 0 |
| Fall 2009 | -P +C | 1 | 0 | 0 |
| Fall 2009 | -P +C | 2 | 0 | 0 |
| Fall 2009 | -P +C | 3 | 0 | 0 |
| Fall 2009 | -P +C | 4 | 0 | 0 |
| Fall 2009 | +P -C | 1 | 3 | 8 |
| Fall 2009 | +P -C | 2 | 2 | 6 |
| Fall 2009 | +P -C | 3 | 10 | 28 |
| Fall 2009 | +P -C | 4 | 0 | 0 |
| Fall 2009 | +P +C | 1 | 11 | 31 |
| Fall 2009 | +P +C | 2 | 11 | 31 |
| Fall 2009 | +P +C | 3 | 10 | 28 |
| Fall 2009 | +P +C | 4 | 1 | 3 |
| Spring 2010 | -P -C | 1 | 0 | 0 |
| Spring 2010 | -P -C | 2 | 0 | 0 |
| Spring 2010 | -P -C | 3 | 0 | 0 |
| Spring 2010 | -P -C | 4 | 0 | 0 |
| Spring 2010 | -P +C | 1 | 0 | 0 |
| Spring 2010 | -P +C | 2 | 0 | 0 |
| Spring 2010 | -P +C | 3 | 0 | 0 |
| Spring 2010 | -P +C | 4 | 0 | 0 |
| Spring 2010 | +P -C | 1 | 10 | 28 |
| Spring 2010 | +P -C | 2 | 17 | 47 |
| Spring 2010 | +P -C | 3 | 9 | 25 |
| Spring 2010 | +P -C | 4 | 1 | 3 |
| Spring 2010 | +P +C | 1 | 51 | 142 |
| Spring 2010 | +P +C | 2 | 83 | 231 |
| Spring 2010 | +P +C | 3 | 51 | 142 |
| Spring 2010 | +P +C | 4 | 1 | 3 |
| Fall 2010 | -P -C | 1 | 0 | 0 |
| Fall 2010 | -P -C | 2 | 0 | 0 |
| Fall 2010 | -P -C | 3 | 0 | 0 |
| Fall 2010 | -P -C | 4 | 0 | 0 |
| Fall 2010 | -P +C | 1 | 0 | 0 |
| Fall 2010 | -P +C | 2 | 0 | 0 |
| Fall 2010 | -P +C | 3 | 0 | 0 |
| Fall 2010 | -P +C | 4 | 0 | 0 |
| Fall 2010 | +P -C | 1 | 5 | 14 |
| Fall 2010 | +P -C | 2 | 7 | 19 |
| Fall 2010 | +P -C | 3 | 10 | 28 |
| Fall 2010 | +P -C | 4 | 0 | 0 |
| Fall 2010 | +P +C | 1 | 35 | 97 |
| Fall 2010 | +P +C | 2 | 28 | 78 |
| Fall 2010 | +P +C | 3 | 24 | 67 |
| Fall 2010 | +P +C | 4 | 0 | 0 |
| Spring 2011 | -P –C | 1 | 0 | 0 |
| Spring 2011 | -P –C | 2 | 0 | 0 |
| Spring 2011 | -P –C | 3 | 0 | 0 |
| Spring 2011 | -P –C | 4 | 0 | 0 |
| Spring 2011 | -P +C | 1 | 0 | 0 |
| Spring 2011 | -P +C | 2 | 0 | 0 |
| Spring 2011 | -P +C | 3 | 0 | 0 |
| Spring 2011 | -P +C | 4 | 0 | 0 |
| Spring 2011 | +P -C | 1 | 5 | 14 |
| Spring 2011 | +P -C | 2 | 22 | 61 |
| Spring 2011 | +P -C | 3 | 6 | 17 |
| Spring 2011 | +P -C | 4 | 0 | 0 |
| Spring 2011 | +P +C | 1 | 15 | 42 |
| Spring 2011 | +P +C | 2 | 25 | 69 |
| Spring 2011 | +P +C | 3 | 8 | 22 |
| Spring 2011 | +P +C | 4 | 0 | 0 |
| Fall 2011 | -P –C | 1 | 0 | 0 |
| Fall 2011 | -P –C | 2 | 0 | 0 |
| Fall 2011 | -P –C | 3 | 0 | 0 |
| Fall 2011 | -P –C | 4 | 0 | 0 |
| Fall 2011 | -P +C | 1 | 0 | 0 |
| Fall 2011 | -P +C | 2 | 0 | 0 |
| Fall 2011 | -P +C | 3 | 0 | 0 |
| Fall 2011 | -P +C | 4 | 0 | 0 |
| Fall 2011 | +P -C | 1 | 7 | 19 |
| Fall 2011 | +P -C | 2 | 1 | 3 |
| Fall 2011 | +P -C | 3 | 8 | 22 |
| Fall 2011 | +P -C | 4 | 0 | 0 |
| Fall 2011 | +P +C | 1 | 6 | 17 |
| Fall 2011 | +P +C | 2 | 2 | 6 |
| Fall 2011 | +P +C | 3 | 5 | 14 |
| Fall 2011 | +P +C | 4 | 0 | 0 |
| Spring 2012 | -P –C | 1 | 0 | 0 |
| Spring 2012 | -P –C | 2 | 0 | 0 |
| Spring 2012 | -P –C | 3 | 0 | 0 |
| Spring 2012 | -P –C | 4 | 0 | 0 |
| Spring 2012 | -P +C | 1 | 0 | 0 |
| Spring 2012 | -P +C | 2 | 0 | 0 |
| Spring 2012 | -P +C | 3 | 0 | 0 |
| Spring 2012 | -P +C | 4 | 0 | 0 |
| Spring 2012 | +P -C | 1 | 0 | 0 |
| Spring 2012 | +P -C | 2 | 0 | 0 |
| Spring 2012 | +P -C | 3 | 5 | 14 |
| Spring 2012 | +P -C | 4 | 0 | 0 |
| Spring 2012 | +P +C | 1 | 1 | 3 |
| Spring 2012 | +P +C | 2 | 1 | 3 |
| Spring 2012 | +P +C | 3 | 5 | 14 |
| Spring 2012 | +P +C | 4 | 0 | 0 |
| Fall 2012 | -P –C | 1 | 0 | 0 |
| Fall 2012 | -P –C | 2 | 0 | 0 |
| Fall 2012 | -P –C | 3 | 0 | 0 |
| Fall 2012 | -P –C | 4 | 0 | 0 |
| Fall 2012 | -P +C | 1 | 0 | 0 |
| Fall 2012 | -P +C | 2 | 0 | 0 |
| Fall 2012 | -P +C | 3 | 0 | 0 |
| Fall 2012 | -P +C | 4 | 0 | 0 |
| Fall 2012 | +P -C | 1 | 0 | 0 |
| Fall 2012 | +P -C | 2 | 0 | 0 |
| Fall 2012 | +P -C | 3 | 5 | 14 |
| Fall 2012 | +P -C | 4 | 0 | 0 |
| Fall 2012 | +P +C | 1 | 1 | 3 |
| Fall 2012 | +P +C | 2 | 1 | 3 |
| Fall 2012 | +P +C | 3 | 5 | 14 |
| Fall 2012 | +P +C | 4 | 0 | 0 |
