# Supplementary material for: Correction: Interactive Effects of Black-Tailed Prairie Dogs and Cattle on Shrub Encroachment in a Desert Grassland Ecosystem
Source: PLoS One. 2016 Jun 9;11(6):e0157688. doi: 10.1371/journal.pone.0157688 (PMC4900552; doi:10.1371/journal.pone.0157688)
Supplement: S1 File — (PDF) [file pone.0157688.s001.pdf]

RESEARCH ARTICLE

Interactive Effects of Black-Tailed Prairie Dogs and Cattle on Shrub Encroachment in a Desert Grassland Ecosystem

Eduardo Ponce-Guevara[1]*, Ana Davidson[2,3], Rodrigo Sierra-Corona[1], Gerardo Ceballos[1]

1 Instituto de Ecología, Universidad Nacional Autónoma de México, México City, México, **2** Department of Ecology and Evolution, Stony Brook University, Stony Brook, New York, United States of America, **3** Department of Biology, University of New Mexico, Albuquerque, New Mexico, United States of America

* eponce@ecologia.unam.mx

CrossMark
click for updates

OPEN ACCESS

Citation: Ponce-Guevara E, Davidson A, Sierra-Corona R, Ceballos G (2016) Interactive Effects of Black-Tailed Prairie Dogs and Cattle on Shrub Encroachment in a Desert Grassland Ecosystem. PLoS ONE 11(5): e0154748. doi:10.1371/journal.pone.0154748

Editor: Kurt O. Reinhart, USDA-ARS, UNITED STATES

Received: March 19, 2014

Accepted: April 18, 2016

Published: May 4, 2016

Copyright: © 2016 Ponce-Guevara et al. This is an open access article distributed under the terms of the Creative Commons Attribution License, which permits unrestricted use, distribution, and reproduction in any medium, provided the original author and source are credited.

Data Availability Statement: Data is available as supporting information.

Funding: This research was partially funded by NSF (Grant OISE-0653296), Consejo Nacional de Ciencia y Tecnología (CONACyT), National University of Mexico (UNAM), the J. M. Kaplan Fund, the Whitley Fund for Nature and the World Wildlife Fund and Carlos Slim Fund Alliance. The funders had no role in study design, data collection and analysis, decision to publish, or preparation of the manuscript.

Abstract

The widespread encroachment of woody plants throughout the semi-arid grasslands in North America has largely resulted from overgrazing by domestic livestock, fire suppression, and loss of native large and small mammalian herbivores. Burrowing-herbivorous mammals, such as prairie dogs (*Cynomys* spp.), help control shrub encroachment through clipping of shrubs and consumption of their seedlings, but little is known about how this important ecological role interacts with and may be influenced by co-existing large herbivores, especially domestic livestock. Here, we established a long-term manipulative experiment using a 2 × 2 factorial design to assess the independent and interactive effects of black-tailed prairie dogs (*Cynomys ludovicianus*) and cattle (*Bos taurus*) on honey mesquite (*Prosopis glandulosa*) abundance and structure. We found that, after five years, mesquite abundance was three to five times greater in plots where prairie dogs were removed compared to plots where they occurred together or alone, respectively. While both prairie dogs and cattle reduced mesquite cover, the effect of prairie dogs on reducing mesquite abundance, cover, and height was significantly greater than that by cattle. Surprisingly, cattle grazing enhanced prairie dog abundance, which, in turn, magnified the effects of prairie dogs on mesquite shrubs. Mesquite canopy cover per hectare was three to five times greater where prairie dogs and cattle were absent compared to where they occurred together or by themselves; whereas, cumulative mesquite height was two times lower on sites where prairie dog and cattle occurred together compared to where they occurred alone or where neither occurred. Data from our experimental study demonstrate that prairie dogs and moderate grazing by cattle can suppress mesquite growth, and, when their populations are properly managed, they may interact synergistically to significantly limit mesquite encroachment in desert grasslands.

Competing Interests: The authors have declared that no competing interests exist.

Introduction

Grasslands are one of the most ubiquitous biomes on the planet, but are highly threatened by intensive land-use activities and conversion into shrublands. The conversion of grasslands into shurblands is associated with increased desertification and the loss of ecosystem services [1]. Desertification of grasslands often results from overgrazing by poorly managed livestock, combined with drought that causes extensive soil erosion, decline in native perennial grass cover, and invasion by woody plants [2–6]. Loss of grasslands to shrubands has been further facilitated by the widespread decline of native, free-roaming, large herbivores (i.e. bison) and small to medium-sized burrowing mammals (i.e. prairie dogs) that both help maintain the presence of the world's grasslands through their herbivory and direct destruction of woody plants [4,5,7–13].

Indeed, large herbivores and burrowing mammals play important roles in shaping the structure and function of grassland ecosystems. Through their grazing, browsing and soil disturbances they transform grassland landscapes, create important habitats for many other grassland species, and enhance spatial and temporal heterogeneity [8,14–16]. In the central grasslands in North America for example, prairie dogs have co-existed with bison for thousands of years and have established important grazing associations. The grazing and burrowing activities by prairie dogs results in more nutritious forage on colony sites that attracts large herbivores like bison [17–19], while grazing by large herbivores benefits prairie dogs by increasing forage quality and reducing vegetation height, thereby increasing predator detection and, consequently, prairie dog survival [17,18,20]. However, domestic cattle (*Bos taurus*) have supplanted native bison (*Bison bison*) throughout most of their historical geographic range, while prairie dogs (*Cynomys* spp.) have declined across 98% of their former range [8,9,19,21]. The widespread decline in bison and prairie dogs, and overgrazing by livestock has been accompanied by a decline in biodiversity and woody plant encroachment into North America's grasslands [6,22–24]. In the desert grasslands of the American Southwest and northern Mexico prairie dogs may control the establishment of honey mesquite (*Prosopis glandulosa*) through their clipping of the mesquite shrubs and consumption of their seedlings [13].

Whereas, poorly managed cattle overgraze and help spread mesquite seeds across the desert grassland landscape [5,6,24–26], and prairie dogs may control their establishment, a major conservation and economic question in rangeland management is whether cattle and prairie dogs can co-exist in a way that supports the needs of local ranching communities, and grassland biodiversity. Our previous work in the desert grasslands of northern Mexico has shown that prairie dogs and cattle can have synergistic impacts on desert grassland vegetation, and that cattle graze preferentially along prairie dog colony edges and use the colony centers for resting, while prairie dog abundance increases in areas grazed by cattle [11,27]. These observations support the argument that prairie dog colonies are not only an important component of the grassland mosaic for maintaining biodiversity, but also may provide valuable habitat for domestic livestock [27]. In fact, the two herbivores can have mutualistic grazing associations, similar to those between bison and prairie dogs [18,19,28]. Yet, how the interactions between prairie dogs and cattle impact mesquite encroachment in desert grasslands remains poorly understood. Insights into this ecological relationship are necessary to understand how traditional management strategies in this environment, such as prairie dog eradication and cattle grazing practices, affect mesquite encroachment. Using a long-term manipulative experiment, we tested the effects of prairie dogs and cattle on the abundance and structure of mesquite shrubs and the effect of cattle on prairie dog abundance. This information, along with improved livestock grazing practices (e.g., moderate grazing), can be used to inform conservation strategies aimed at limiting the transition from grassland to shrubland and promoting the co-existence of native burrowing mammals and large, domestic herbivores.

Materials and Methods

Study site

We conducted our experiment within the Janos Biosphere Reserve in the northwestern region of Chihuahua, Mexico, located 75 km south of United States-Mexico border. The study site is located on the Nature Conservancy´s El Uno Ecological Reserve (Private Lands Program, Mexico), within the *Báscula* prairie dog colony (30°54´N 108°26´W; Fig 1). Before the acquisition of the property, the site was grazed by cattle for but in 2004 cattle were removed to allow vegetation recovery. Coupled with extensive livestock overgrazing, the region has undergone a shift from a perennial grassland to what is now largely annual grassland [24,29]. The study site is in a broad basin, with a sandy loam soil surface texture and sandy clay loam sub-surface. Vegetation is dominated by the annual grasses, sixweeks threeawn (*Aristida adscensionis*), needle grama (*Bouteloua aristidoides*), and sixweeks grama (*B. barbata*), and numerous forbs. Perennial grasses present include poverty threeawn (*Aristida divaricata*), ear muhly (*Muhlenbergia arenacea*), vine mesquite (*Panicum obtusum*), and tobosagrass (*Pleuraphis mutica*), with some blue grama (*B. gracilis*). The area experiences a wide inter-seasonal temperature variation, from 42°C in summer to -10°C, in winter and a mean annual temperature of 16.9°C. Mean annual precipitation is 306 mm and most of the precipitation falls during the summer monsoon period [11,29].

Experimental design

In 2006, we established four replicate experimental blocks consisting of sixteen 60 × 60 m plots (0.36 ha each) in an area with similar soil type, plant species composition, and prairie dog densities (Fig 1A). Each plot was separated by 10 m. The study site had not been grazed by cattle for two years prior to the initiation of the study. Each block, separated by 30m, had the following 2 × 2 factorial design: both prairie dogs and cattle (+P+C); only prairie dogs (+P-C); only cattle (-P+C); and both species absent (-P-C). Distance among blocks ranged between 50 m and 150 m [11].

Prairie dog treatment

Prairie dog enclosures were installed during the second year of the study. Initially, prairie dogs were present on all plots, and the -P+C and -P-C treatments were implemented by trapping and relocating prairie dogs to elsewhere on the REU. We prevented their recolonization by fencing the plots with 2.54 cm poultry netting that still allowed access by other small mammals (Fig 1B). The wire-mesh extended 0.70 m aboveground, and was buried 1.25 m below the soil surface to deter prairie dogs from burrowing underneath. A 15.24 cm wide strip of metal flashing was attached along the top of the poultry-wire to prevent prairie dogs from climbing over the fences. In plots with prairie dogs, we counted prairie dogs for two consecutive mornings (7:00 to 10:00 hrs), during spring (last week of March) and fall (second week of September) of each year: from 2006 (baseline pretreatment) through 2007, 2008, 2009, 2010, and 2011.

Ethics statement

Trapping and translocation were carried out in strict accordance with the recommendations of the Department of Veterinary Medicine of the National Autonomous University of Mexico (UNAM). Capture and handling protocols were reviewed and authorized by the Mexican Wildlife Department (Permit Number: SGPA/DGVS/0844/06). All permits required for prairie dog capture and handling were requested from and authorized by the administration of REU. Efforts were made to minimize prairie dog suffering during the capture and relocation procedures.

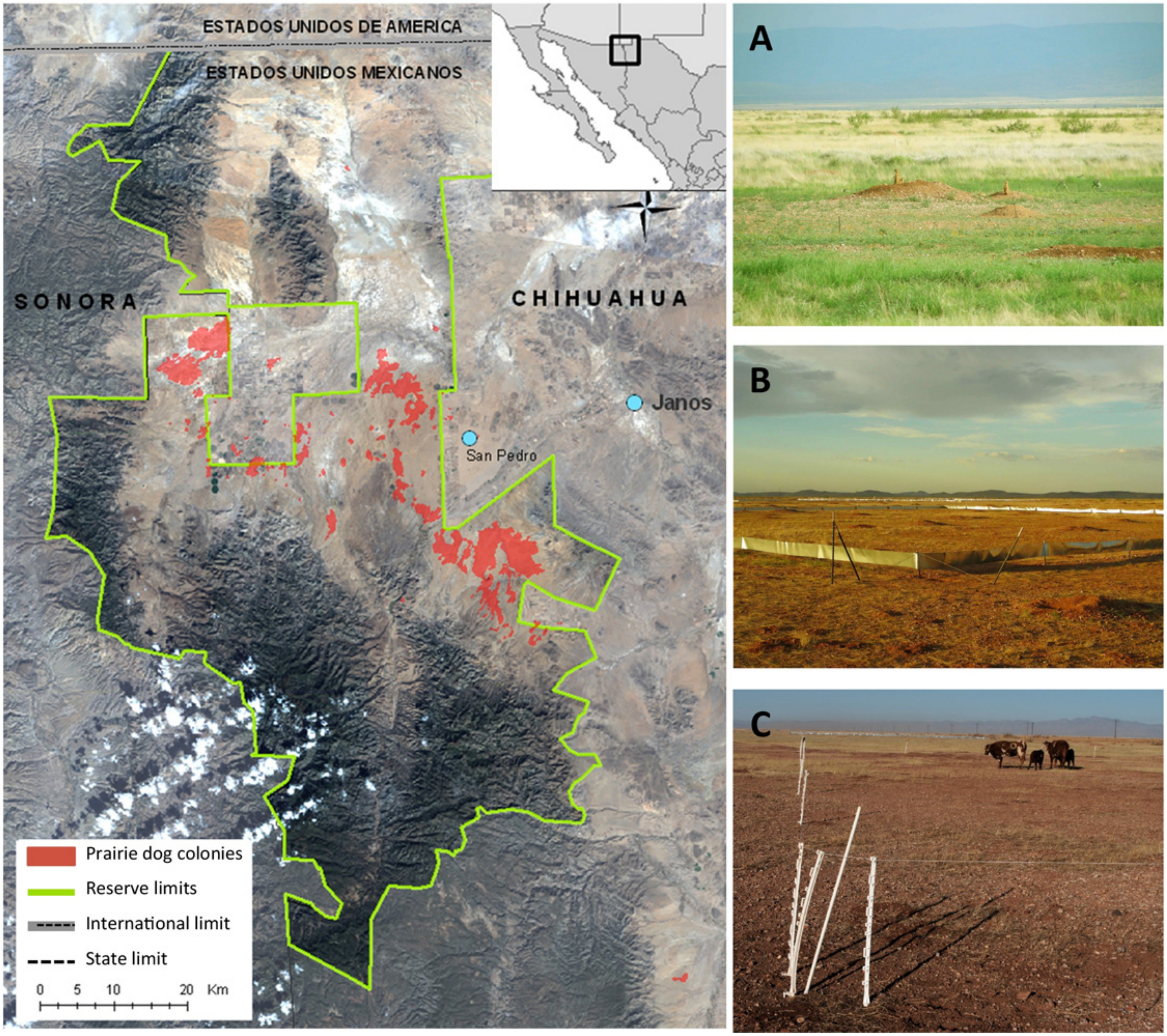

Fig 1. Study site. The Nature Conservancy´s El Uno Ecological Reserve is located within the Janos Biosphere Reserve. Reprinted from Landsat Satellite Images (LC80340392015154LGNL00 and LC803340392015154LGN00) under a CC BY license, data courtesy of the US Geological Survey. Using a 2 × 2 factorial design, our experimental plots consisted of: both prairie dogs and cattle (+P+C); only prairie dogs (+P-C); only cattle (-P+C); and both species absent (-P-C). (A) Plots were established in sites with similar soil type, plant species composition, and prairie dog densities. (B) Prairie dogs were trapped and removed from experimental plots (-P-C, -P+C) and their exclusion was maintained using fencing that extended above and belowground. (C) Cattle were confined within plots (+P+C, -P+C) using an electric fence during the winter period to achieve a conservative grazing regime (consumption of 40% of available forage).

doi:10.1371/journal.pone.0154748.g001

Cattle treatment

In treatments where cattle occurred (+P+C, -P+C), cattle were placed in the plots to simulate a conservative grazing regime [11]. For this experiment, a conservative grazing regime was defined as the consumption of 40% of forage (i.e., the available plant biomass) by cattle during

winter [25]. Available forage was estimated in each plot every winter. Crossbred beef cows were used to remove 40% of the available plant biomass. Cows were allowed to graze for 12 hrs and were contained within the study plots using electric fencing (Fig 1C). The number of cows per plot varied on each plot depending on available forage, and similarly, the number of cattle varied across years due to environmental conditions and plant production. Beef cows were primarily British (Angus, Hereford) and Continental (Limousin and Charolais) breeds raised on nearby pastures (ejido San Pedro, Rancho San Blas, Rancho La Soledad).

Ethics statement

Transportation and manipulation of domestic cattle was conducted according to the Mexican Official Norm (NOM-051-ZOO-1995), which deals with humanitarian treatment of animal mobilization [30]. Trained technicians handled the cattle to guarantee cattle safety. No official permit was necessary to perform this experiment because domestic cattle under extensive production are not considered experimental animal species under the Official Mexican Norm (NOM-062-ZOO-1999), which provide technical specifications for the production, care and use of lab animals. Cattle owners from the local community agreed to collaborate with the project by providing their cattle during the experiment.

Mesquite abundance and structure

We assessed mesquite abundance (individuals per hectare) in each plot during the summers of 2006 and 2011 by counting all mesquite plants and classifying each individual plant as adult or seedling. To evaluate the effect of prairie dogs and cattle on the structure of mesquite, we also measured total canopy cover and height of mesquite shrubs within each of the treatment plots. To determine mesquite shrub canopy area per plot, we collected two horizontal measurements of each plant in the summer of 2011 (no cover data were collected in 2006): the longest horizontal canopy width and the corresponding perpendicular canopy width. We calculated average individual shrub canopy (m^2) for each plot and then multiplied this by mesquite abundance (ind/ha) within each plot to obtain a comparative measure of cover per treatment (m^2 of mesquite canopy per hectare). To determine the effect of prairie dogs and cattle on individual mesquite height, we estimated cumulative (i.e. total) mesquite height per ha. To control for varying mesquite densities and sizes per unit area, average mesquite height was multiplied by abundance (ind/ha) to produce an estimate of cumulative mesquite height (cm per ha), henceforth referred to as "mesquite height".

Data analysis

We assessed all data for normality, and if needed, normalized data by log transformations (Infostat Statistical Package, V. 2011). We conducted a Repeated Measure Analysis of Variance (RMANOVA) to test the effects of treatments over time on mesquite abundance, and an ANOVA to evaluate differences in canopy cover and height among treatments.

Results

Effects of prairie dogs and cattle on mesquite shrubs

Mesquite abundance increased significantly in plots where prairie dogs were removed (-P-C, -P+C), and the increase was greatest (3-fold increase) in plots where neither prairie dogs nor cattle occurred (-P-C) (RMANOVA: Wilks, $\lambda = 0.24$, $F_{6,22} = 3.76$, $P = 0.001$). Whereas, mesquite abundance did not change over time in plots with prairie dogs (+P-C, +P+C); these plots maintained the lowest abundance of mesquite shrubs across treatments (Fig 2 and S1 Table).

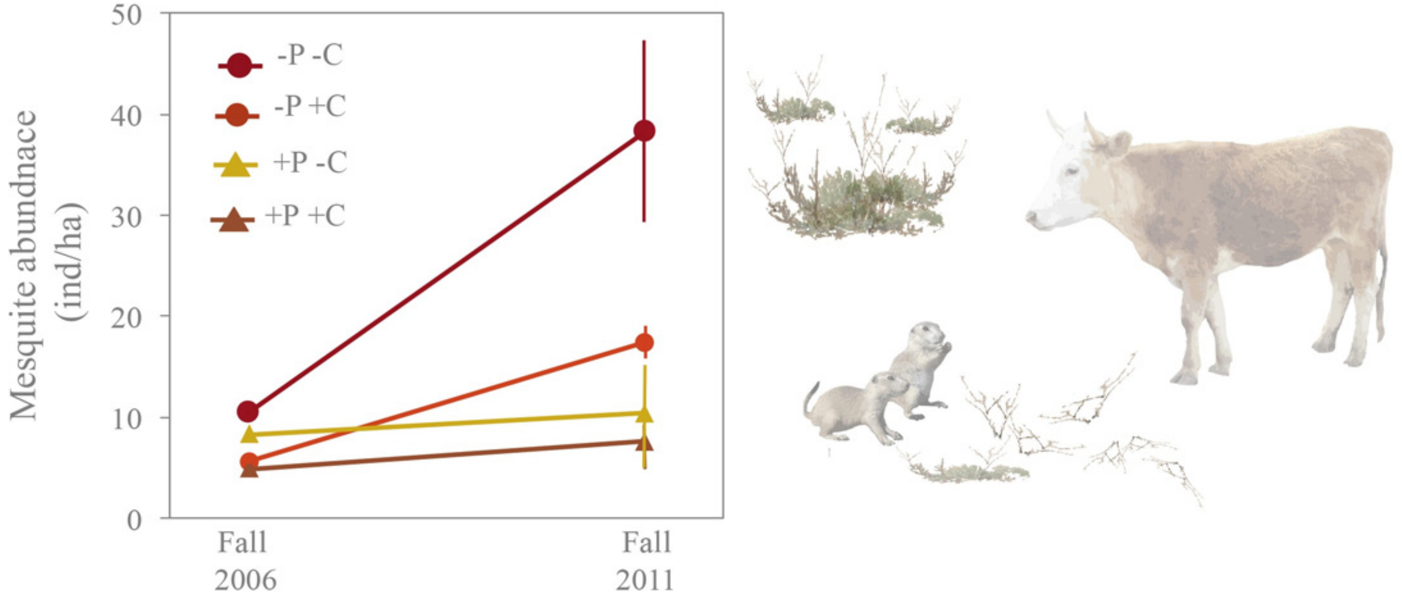

Fig 2. Honey mesquite and black-tailed prairie dog abundance. Prairie dogs and cattle significantly impacted mesquite abundance. After five years of implementing our experimental treatments, mesquite abundance (mean ± SD) increased in plots that excluded prairie dogs (-P-C, -P+C) but remained lowest and unchanged in plots with prairie dogs (+P+C, +P-C). (+P+C = prairie dogs and cattle occurred together; +P-C = prairie dogs only occurred; -P+C = cattle only occurred; -P-C = both prairie dog and cattle were absent)

doi:10.1371/journal.pone.0154748.g002

Our results show that mesquite cover was five times greater where neither prairie dogs nor cattle were present, compared to where they occurred by themselves and where both were present (ANOVA: $F_{3,15} = 4.857$, P = 0.004; Fig 3 and S2 Table).

Mesquite height was also greatest on plots where neither species was present, and lower in plots with prairie dogs (ANOVA: $F_{3,15} = 6.6163$, P = 0.0004, Fig 4A and S3 Table). Compared to where all animals were removed (-P-C), we found that individual mesquite height was 13% lower in on plots where only prairie dogs were present (+P-C), 15% lower in on plots where only cattle were present (-P+C), and surprisingly 46% lower in plots where both prairie dogs and cattle were present (+P+C) (Fig 4A and S2 Table). The magnitude of treatment effects were more obvious when comparing estimates of total mesquite height per unit area (m per ha) (Fig 4B). Specifically, plots without both prairie dogs and cattle had the highest cumulative

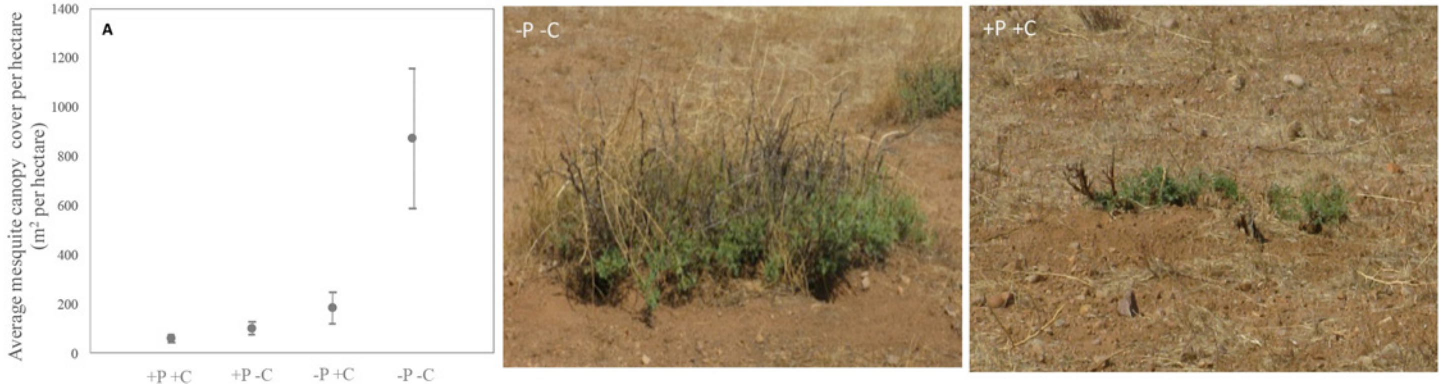

Fig 3. Mesquite canopy cover response to experimental treatments. Average mesquite canopy cover (mean ± SD) per hectare on each treatment. Canopy cover was strikingly five-fold greater in plots where neither species was present compared to where they occurred alone or together (F STAT, P < 0.0004). (+P+C = prairie dogs and cattle present; +P-C = prairie dogs present; -P+C = cattle present; -P-C = both species absent)

doi:10.1371/journal.pone.0154748.g003

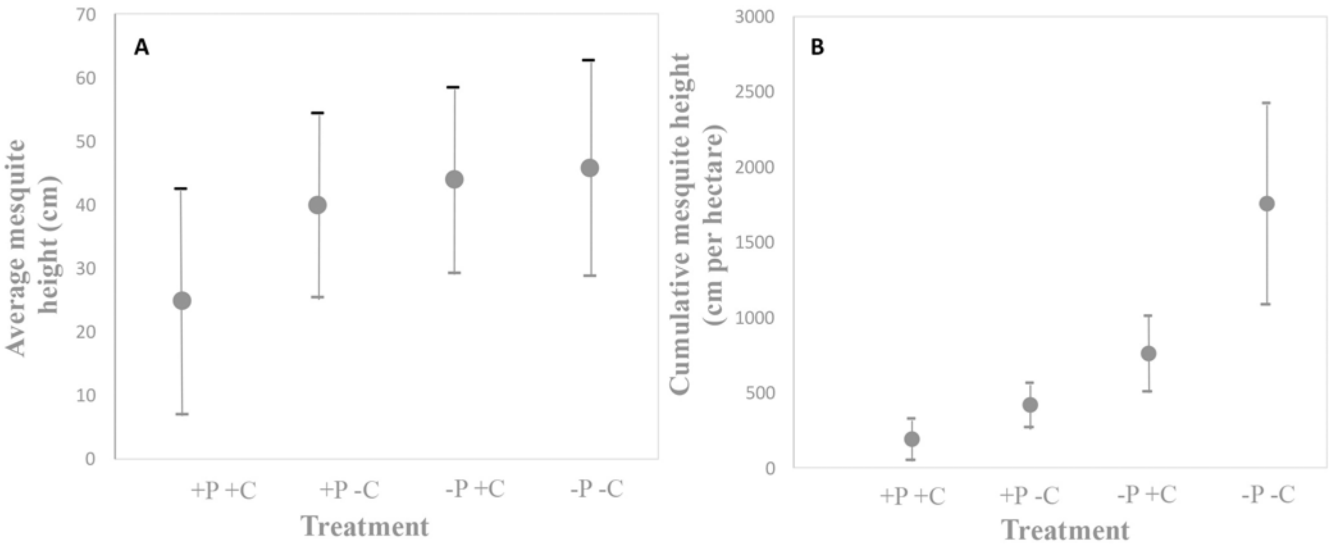

Fig 4. Mesquite height response to experimental treatments. (A) Effect of treatments on average mesquite height, and (B) cumulative mesquite height. Treatments had minimal effects on average mesquite height (A) but large effects on cumulative height (B) (+P+C = prairie dogs and cattle present; +P-C = prairie dogs present; -P+C = cattle present; -P-C = both species absent)

doi:10.1371/journal.pone.0154748.g004

height, whereas in the plots where prairie dogs and cattle occurred alone or together, their herbivory pressures considerably reduced cumulative mesquite height.

The effect of cattle on prairie dog abundance

One year after treatments, the number of prairie dogs was consistently higher on plots grazed by cattle compared to plots without cattle (Fig 5 and S3 Table). These differences were consistent across the study from 2007 through 2011, and were significant in the spring periods of

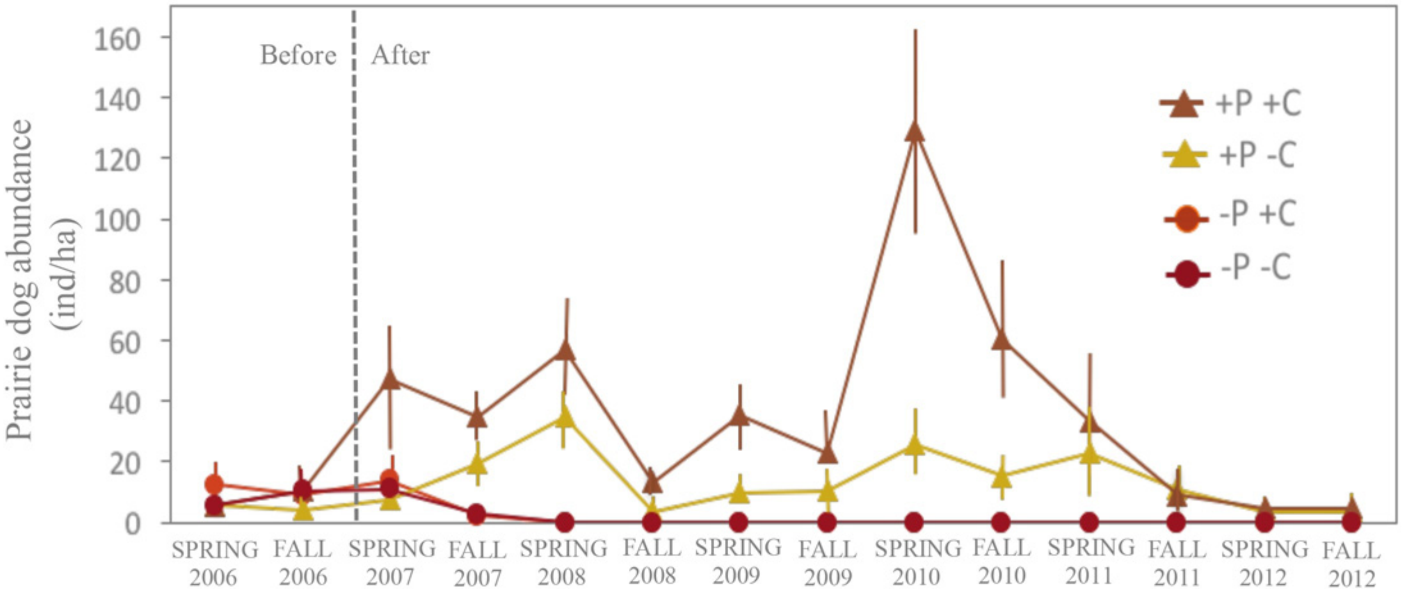

Fig 5. Prairie dog abundance. Number of prairie dogs (mean ± SD per ha) on each treatment, based on number of animals that occurred on each plot. +P+C = prairie dogs and cattle occurred together; +P-C = prairie dogs only occurred; -P+C = cattle only occurred; -P-C = both prairie dog and cattle were absent

doi:10.1371/journal.pone.0154748.g005

2007 and 2009 and fall periods of 2008, 2010, and 2011 ($P < 0.05$; S3 Table). Prairie dog abundance was likely also influenced by annual precipitation and variation in vegetation production. The study site received only 65% and 45% of the historical average annual rainfall in 2009 and 2011, respectively, and during those years prairie dog abundance decreased across all plots and no differences in their abundances were observed among treatments. Conversely, rainfall was 10% above historic values during 2010 when maximum prairie dog abundance occurred during the study period.

Discussion and Conclusion

Effects of prairie dogs and cattle on mesquite

Our study demonstrates that prairie dogs and cattle, together, can reduce mesquite shrub invasion in the desert grasslands of northern Mexico. The removal of prairie dogs had a striking effect on mesquite shrub abundance, cover, and height, and this effect was notably much greater when cattle were also absent (Figs 2, 3 and 4). Consistent with previous studies [13,24], our experiment demonstrates the important role of prairie dogs in controlling mesquite through their foraging and clipping activities. In other parts of the world, small to medium-sized herbivorous mammals also affect shrub establishment [9]. For example, plains vizcachas (*Lagostomus maximus*) in the Argentinian Pampas, burrowing bettongs (*Bettongia lesueur*) in Australia, and kangaroo rats (*Dipodomys* spp.) in North America reduce shrub encroachment through browsing of shrubs and consumption of seeds and seedlings [8,9,31,32].

Cattle have been a major disperser of mesquite seeds in semi–arid grasslands, and poorly managed cattle are well-known to overgraze and desertify grasslands [6,23,33]. However, our experiment demonstrates that cattle also suppress mesquite abundance through: 1) their direct herbivory; and 2) moderate grazing that increases the populations of prairie dogs (Fig 5), which enhances the ecological service of prairie dogs in the suppression of mesquite shrubs. Grazing associations between prairie dogs and cattle can be positive, neutral or negative depending on spatial and temporal variability of grassland ecosystems [34], but our work indicates that their positive grazing association can be capitalized on to strategically reduce shrub invasion.

Management implications

Despite little direct evidence, competition with cattle has been used to justify extensive programs to eradicate prairie dogs from grasslands, perceptions on prairie dogs have been pushed to extremes. These prairie dog "pest control" programs have been a major cause for reducing prairie dog populations to about 2% of their historic numbers and still continue today [35]. However, such eradication efforts have been counterproductive, resulting in invasion of woody plants, like mesquite, into grasslands. Indeed, there now is strong evidence from multiple experimental and long-term studies that prairie dogs play an important role in controlling shrub encroachment [13,24]. If the main challenge is to maintain grasslands and livestock production then scientists and managers need to work together to find productive ways to improve livestock management, such as through moderate grazing practices and/or by restoring prairie dog populations in rangeland ecosystems to help control shrub encroachment and recover native grasslands.

The results of our experiment demonstrated the important, interactive effects of prairie dogs and cattle on mesquite encroachment in desert grasslands. However, our multi-year experiment also indicates considerable temporal variation in prairie dog abundance, driven by changes over time in precipitation (or plant biomass). Bottom-up regulation of rodent populations are common in arid ecosystems [36,37], and our results similarly show that bottom-up

drivers regulate the ecological association and interactive effects of prairie dogs and cattle. For example, our data suggests that cattle in desert grasslands have more pronounced effects on facilitating prairie dog colony expansion during wet years when the vegetation is tall and prairie dog abundance is high. In contrast, the role of cattle in facilitating the expansion of prairie dog populations may be less important during dry years when productivity of grassland vegetation is low and consequently the habitat remains more open, and prairie dog densities are lower [20,27,28,38]. More research is needed to improve our understanding of the spatial and temporal variation of their grazing associations and how their associations vary under different grazing regimes in order to better manage arid rangelands [25,34,39]. For example, we used a moderate grazing intensity for our experiment, where cattle grazed 40% of available winter forage and showed positive and synergistic effects with prairie dogs on grassland composition and structure [25]. Yet, high levels of grazing intensity and season long-grazing are common in the area [24,29]. In conclusion, we believe that reintroducing prairie dogs and expanding their populations will not restore desert grasslands without also improving overall livestock grazing management. Scientific data are needed to develop more effective conservation and management strategies for the desert grasslands of northern Mexico, and our long-term experiment provides insights into a novel approach that can be strategically used to help achieve this goal.

Supporting Information

S1 Table. Mesquite abundance database. Number of mesquite shrubs (ind/plot and ind/hectare) observed in 2006 and 2011.
(DOCX)

S2 Table. Mesquite height and canopy cover database. Mesquite shrubs observed and measured in 2011.
(DOCX)

S3 Table. Prairie dog database. Number of prairie dogs observed (ind/plot and ind/hectare) from 2006 to 2011.
(DOCX)

Acknowledgments

This paper has been submitted as a partial fulfillment of the graduate program in biological sciences at the National Autonomous University of Mexico (UNAM). We express our sincere gratitude to the following individuals for their support: Ed Fredrickson and David Lightfoot for their valuable advice on the design of the study; Ed Fredrickson for his assistance and guidance on the cattle component of our experiment; Jesus Pacheco, Ana Montiel, Daniel Aceves, and Jorge Díaz for their invaluable help with field data collection; Karla Pelz, Jose González-Maya, and Rodrigo Medellín for their comments during the early stages of the manuscript; Rosalba Berecerra for support in figure design; Lourdes Martínez-Estévez for the illustrations; and Laura Paulson, Antonio Esquer, and Nelida Barajas (TNC Private Land Program) for letting us work in the Rancho El Uno and for the use of the facilities during the study. We also thank Roland Bjorkland for help with editing the manuscript.

Author Contributions

Conceived and designed the experiments: EPG AD GC. Performed the experiments: EPG AD RSC. Analyzed the data: EPG AD. Wrote the paper: EPG AD GC.

References

1. Suttie JM, Reynolds SG, Botello C. editors. Grasslands of the world. Rome, Italy: Food and Agriculture Organization of the United Nations. 2005.
2. Schlesinger WH, Reynolds JF, Cunningham GL, Huenneke LF, Jarrell WM, Virginia RA, et al. Biological feedbacks in global desertification. Science. 1990; 247: 1043–8. doi: 10.1126/science.247.4946.1043 PMID: 17800060
3. Geist HJ, Lambin EF. Dynamic Causal Patterns of Desertification. Bioscience. 2004; 54: 817–29. doi: 10.1641/0006-3568(2004)054[0817:DCPOD]2.0.CO;2
4. Roques KG, O'Connor TG, Watkinson AR. Dynamics of shrub encroachment in an African savanna: relative influences of fire, herbivory, rainfall and density dependence. J Appl Ecol. 2001; 38: 268–280. doi: 10.1046/j.1365-2664.2001.00567.x
5. Briggs JM, Knapp AK, Blair JM, Heisler JL, Hoch GA, Lett MS, et al. An Ecosystem in Transition: Causes and Consequences of the Conversion of Mesic Grassland to Shrubland. Bioscience. 2005; 55: 243–54. doi: 10.1641/0006-3568(2005)055[0243:AEITCA]2.0.CO;2
6. Van Auken OW. Causes and consequences of woody plant encroachment into western North American grasslands. J Environ Manage. 2009; 90: 2931–42. doi: 10.1016/j.jenvman.2009.04.023 PMID: 19501450
7. Pringle RM. Elephants as agents of habitat creation for small vertebrates at the patch scale. Ecology. 2008; 89: 26–33. doi: 10.1890/07-0776.1 PMID: 18376543
8. Delibes-mateos M, Smith AT, Slobodchikoff CN, Swenson JE. The paradox of keystone species persecuted as pests: A call for the conservation of abundant small mammals in their native range. Biol Conserv. 2011; 144: 1335–46. doi: 10.1016/j.biocon.2011.02.012
9. Davidson A, Detling J, Brown J. Ecological roles and conservation challenges of social, burrowing, herbivorous mammals in the world´s grasslands. Front Ecol Environ. 2012; 10: 477–85. doi: 10.1890/110054
10. Burns C, Collins S, Smith M. Plant community response to loss of large herbivores: comparing consequences in a South African and a North American grassland. Biodivers Conserv. 2009; 2327–42. doi: 10.1007/s10531-009-9590-x
11. Davidson AD, Ponce E, Lightfoot DC, Fredrickson EL, Brown JH, Cruzado J, et al. Rapid response of a grassland ecosystem to an experimental manipulation of a keystone rodent and domestic livestock. Ecology. 2010; 91: 3189–200. Available: http://www.ncbi.nlm.nih.gov/pubmed/21141180 PMID: 21141180
12. Knapp A. Grassland Dynamics, Long-term Ecological Research in Tallgrass Prairie. Oxford University Press; 1998.
13. Weltzin JF, Archer S, Heitschmidt RK. Small-Mammal Regulation of Vegetation Structure in a Temperate Savanna. Ecology. 1997; 78: 751–63.
14. Allred BW, Fuhlendorf SD, Hamilton RG. The role of herbivores in Great Plains conservation: comparative ecology of bison and cattle. Ecosphere. Ecological Society of America; 2011; 2: art26. doi: 10.1890/ES10-00152.1
15. Fuhlendorf SD, Engle DM. Restoring Heterogeneity on Rangelands: Ecosystem Management Based on Evolutionary Grazing Patterns. Bioscience. 2001; 51: 625–32.
16. Anderson R. Evolution and origin of the Central Grassland of North America: climate, fire, and mammalian grazers. J Torrey Bot Soc. 2006; 133: 626–47. doi: 10.3159/1095-5674(2006)133
17. Coppock JE, Ellis JE, Detling JK, Dyer MI. Plant-Herbivore Interactions in a North American Mixed-Grass Prairie II. Responses of Bison to Modification of Vegetation by Prairie Dogs. Oecologia. 1983; 56: 10–5.
18. Krueger K. Feeding Relationships Among Bison, Pronghorn, and Prairie Dogs: An Experimental Analysis. Ecology. 1986; 67: 760–70.
19. Miller B, Ceballos G, Reading R. The Prairie Dog and Biotic Diversity. Conserv Biol. 1994; 8: 677–81. Available: http://onlinelibrary.wiley.com/doi/10.1046/j.1523-1739.1994.08030677.x/full
20. Lloyd N, Moehrenschlager A, Smith DH, Bender D. Food limitation at species range limits: Impacts of food availability on the density and colony expansion of prairie dog populations at their northern periphery. Biol Conserv. 2013; 161: 110–17. doi: 10.1016/j.biocon.2013.03.008
21. List R, Ceballos G, Curtin C, Gogan PJP, Pacheco J, Truett J. Historic distribution and challenges to bison recovery in the northern Chihuahuan Desert. Conserv Biol. 2007; 21: 1487–94. doi: 10.1111/j.1523-1739.2007.00810.x PMID: 18173472
22. Reviews A, Review A. SHRUB INVASIONS OF NORTH AMERICAN. 2011; 31: 197–215.

23. Knapp AK, Briggs JM, Collins SL, Archer SR, Bret-Harte MS, Ewers BE, et al. Shrub encroachment in North American grasslands: shifts in growth form dominance rapidly alters control of ecosystem carbon inputs. Glob Chang Biol. 2008; 14: 615–623. doi: 10.1111/j.1365-2486.2007.01512.x

24. Ceballos G, Davidson A, List R, Pacheco J, Manzano-Fischer P, Santos-Barrera G, et al. Rapid decline of a grassland system and its ecological and conservation implications. PLoS One. 2010; 5: e8562. doi: 10.1371/journal.pone.0008562 PMID: 20066035

25. Holechek JL, Baker TT, Boren JC, Galt D, Holechek BJL. Grazing Impacts on Rangeland Vegetation: What We Have Learned? Rangelands. 2006; 28: 7–13. doi: http://dx.doi.org/10.2111/1551-501X(2006)28.1[7:GIORVW]2.0.CO;2

26. Freilich JE, Emlen JM, Duda JJ, Freeman DC, Cafaro PJ. Ecological Effects of Ranching: A Six-Point Critique. Bioscience. 2011; 53: 759–765.

27. Sierra–Corona R, Davidson A, Fredrickson EL, Luna-Soria H, Suzan-Azpiri H, Ponce-Guevara E, et al. Black-Tailed Prairie Dogs, Cattle, and the Conservation of North America's Arid Grasslands. PLoS One. 2015; 10: e0118602. doi: 10.1371/journal.pone.0118602 PMID: 25760377

28. Guenther D a, Detling JK. Observations of cattle use of prairie dog towns. J Range Manag. 2003; 56: 410–417. doi: 10.2307/4003830

29. List R, Pacheco J, Ponce E, Sierra-Corona R, Ceballos G. The Janos Biosphere Reserve Northern Mexico. Int J Wilderness. 2010; 16: 35–41. Available: http://ijw.org/wp-content/uploads/2010/05/Aug-2010-IJW-for-WILDSmall.pdf#page=36

30. SAGARPA. NORMA Oficial Mexicana NOM-051-ZOO-1995, Trato humanitario en la movilizacion de animales. Diario Oficial de la Federacion. 1998: 42–67.

31. Branch LC, Hierro JL, Villarreal D. Patterns of plant species diversity following local extinction of the plains vizcacha in semi-arid scrub. 1999; 173–182.

32. Smith AT, Foggin JM. The plateau pika (Ochotona curzoniae) is a keystone species for biodiversity on the Tibetan plateau. 1999; 235–240.

33. Archer S, Scifres C, Bassham CR. Autogenic Succession in a Subtropical Savanna: Conversion of Grassland to Thorn Woodland Author (s): Steve Archer, Charles Scifres, C. R. Bassham, Robert Maggio Reviewed work (s): Published by: Ecological Society of America Stable URL: http://. America (NY). 2012; 58: 111–127.

34. Augustine DJ, Springer TL. Competition and facilitation between a native and a domestic herbivore: trade-offs between forage quantity and quality. Ecol Appl. 2013; 23: 850–63. Available: http://www.ncbi.nlm.nih.gov/pubmed/23865235 PMID: 23865235

35. Miller BJ, Reading RP, Biggins DE, Detling JK, Forrest SC, Hoogland JL, et al. Prairie Dogs: An Ecological Review and Current Biopolitics. J Wildl Manage. 2007; 71: 2801–2810. doi: 10.2193/2007-041

36. Brown JH, Ernest SKM. Rain and Rodents: Complex Dynamics of Desert Consumers. Bioscience. 2002; 52: 979. doi: 10.1641/0006-3568(2002)052[0979:RARCDO]2.0.CO;2

37. Lightfoot DC, Davidson AD, Parker DG, Hernández L, Laundré JW. Bottom-up regulation of desert grassland and shrubland rodent communities: implications of species-specific reproductive potentials. J Mammal. 2012; 93: 1017–1028. doi: 10.1644/11-MAMM-A-391.1

38. Facka AN, Roemer GW, Mathis VL, Kam M, Geffen E. Drought Leads to Collapse of Black-Tailed Prairie Dog Populations Reintroduced to the Chihuahuan Desert. J Wildl Manage. 2010; 74: 1752–1762. doi: 10.2193/2009-208

39. Derner J, Detling J, Antolin M. Are Livestock Weight Gains Affected by Black-Tailed Prairie Dogs? Front Ecol Environ. 2006; 4: 459–464. Available: http://www.esajournals.org/doi/abs/10.1890/1540-9295(2006)4%5B459:ALWGAB%5D2.0.CO%3B2
